# Psychological changes in athletes infected with Omicron after return to training: fatigue, sleep, and mood

**DOI:** 10.7717/peerj.15580

**Published:** 2023-06-15

**Authors:** Chenhao Tan, Jinhao Wang, Guohuan Cao, Yelei He, Jun Yin, Yudan Chu, Zhizhong Geng, Longji Li, Jun Qiu

**Affiliations:** 1 Shanghai Research Institute of Sports Science (Shanghai Anti-Doping Agency), Shanghai, China; 2 Shanghai University of Sport, Shanghai, China

**Keywords:** COVID-19 Omicron strain, Return to training, Fatigue, Sleep, Mood

## Abstract

**Background:**

This study aims to analyze the changes of approximately 1 month in fatigue, sleep, and mood in athletes after returning to training following infection with the COVID-19 Omicron strain and provide recommendations for returning to training after infection.

**Methods:**

Two hundred and thirty professional athletes who had returned to training after being infected with COVID-19 in December 2022 were recruited to participate in three tests conducted from early January 2023. The second test was completed approximately 1 week after the first, and the third was completed about 2 weeks after the second. Each test consisted of completing scales and the exercise-induced fatigue measure. The scales included a visual analog scale, the Athens Insomnia Scale for non-clinical application, and the Depression-Anxiety-Stress scale. The exercise task was a six-minute stair climb test, and athletes evaluated subjective fatigue levels before and after exercise using another Visual Analog Scale and the Karolinska Sleepiness Scale.

**Results:**

After returning to training, athletes’ physical fatigue decreased initially but increased as training progressed. Cognitive fatigue did not change significantly. The exercise task led to elevated levels of physical fatigue after a longer duration of training. Sleep quality problems decreased rapidly after the start of training but remained stable with prolonged training. Depression levels continued to decline, while anxiety levels only reduced after a longer duration of training. Stress levels decreased rapidly after the start of training but did not change with prolonged training.

**Conclusion:**

Athletes who return to training after recovering from COVID-19 experience positive effects on their fatigue, sleep, and mood. It is important to prioritize anxiety assessment and interventions during the short period after returning and to continue monitoring fatigue levels and implementing recovery interventions over a longer period of time.

## Introduction

The COVID-19 pandemic has entered a new phase with the emergence of the highly infectious Omicron strain, which poses a significant health threat due to its pronounced immune breakthrough properties (Zhou, Zhi & Teng, 2023). Following the adjustment of China’s epidemic prevention and control policy in December 2022, infections increased sharply in a short period. Although the exact number of infections during this phase remains unknown, research models have revealed high levels of infection and transmission rates (Goldberg et al., 2023; Leung et al., 2023). Antibody sampling and testing have confirmed high transmission rates during this period (Huang et al., 2023; Liang et al., 2023). Despite the implementation of vaccines and protective measures, athletes who have been well-protected by control measures such as closed-loop management or “bubble” camps for a long period of time were not able to avoid large-scale infections (Su et al., 2022; Washif et al., 2021).

After contracting a COVID-19 infection, timely medical attention and sufficient rest are essential for recovery. However, athletes face the additional challenge of returning to training as soon as possible. It is crucial not only for maintaining athletic performance and fitness levels and preparing for significant events (Hughes et al., 2022; Wilson et al., 2020). To ensure a safe and effective return to play, several studies provided recommendations for guidelines on athletes’ return to training and health monitoring after training (Hughes et al., 2022; Wilson et al., 2020; Lindsay et al., 2021; Elliott et al., 2020; Giusto & Asplund, 2022; McKinney et al., 2021; Phelan et al., 2020; Toresdahl & Asif, 2020). Most researchers have suggested monitoring psychological factors, such as fatigue, sleep, and mood, in addition to symptoms directly related to exercises, such as cardiorespiratory fitness and muscle strength, to ensure safe and effective training after return (Wilson et al., 2020; Lindsay et al., 2021; Giusto & Asplund, 2022; McKinney et al., 2021; Elliott et al., 2022). Therefore, monitoring and intervention of psychological symptoms are key factors in training.

Fatigue, sleep quality, and mood problems such as depression and anxiety are common psychological-related symptoms after COVID-19 infection and are important factors to consider for athletes’ training and athletic performance (Wilson et al., 2020; Lindsay et al., 2021; Giusto & Asplund, 2022; McKinney et al., 2021; Elliott et al., 2022). Among these symptoms, fatigue is the most frequently observed post-infection reaction, including a psychological and a physical component (Arjun et al., 2023; Manu, 2022; Nowakowski et al., 2022; Shen et al., 2023; Stengel et al., 2021). Sleep quality problems are also common in COVID-19 patients, with studies reporting poor sleep patterns, satisfaction, alertness, and efficiency after infection, particularly in patients with long COVID (Alzueta et al., 2022; Merikanto et al., 2022; Scarpelli et al., 2022). Mood problems, such as anxiety and depression, are even more common, are strongly associated with mental health and may even affect the overall pace of rehabilitation (Barker-Davies et al., 2020; Gaber, 2021).

These psychological aspects can significantly influence the safety and effectiveness of an athlete’s training after returning from COVID-19 infection. Both mental or cognitive and physical or strength fatigue are critical components of athletes’ training and athletic performance (Pageaux & Lepers, 2018; Abd-Elfattah, Abdelazeim & Elshennawy, 2015; Almonroeder et al., 2020; Roelands et al., 2021). Sleep is essential for athletes to recover from fatigue, and numerous studies have demonstrated the relationship between sleep quality and athletic performance (Malhotra, 2017; Halson, 2013; Kölling et al., 2019; O’Donnell, Beaven & Driller, 2018). Emotions are closely related to athletes’ motivation and self-efficacy and are essential targets for psychological interventions and training.

Although several guidelines for athletes returning to training have emphasized these psychological factors (Elliott et al., 2020; McKinney et al., 2021), limited research has been conducted to characterize athletes’ performance on these factors after returning to training. Most of the research on psychological factors in athletes under pandemic conditions has focused on the effects of lockdown management or training and competitions suspension and its impact on the sports industry (Fiorilli et al., 2021; Üngür & Karagözoğlu, 2021; Mon-López et al., 2020; Washif et al., 2023).

Several studies have retrospectively examined the differences in depression and anxiety levels between infected and uninfected athletes (Corona et al., 2022; Lima et al., 2022, 2021). Moreover, some researchers have investigated the impact of various factors, such as symptoms, level of athleticism, and type of sport, on athletes’ mental health, fatigue, and other psychological states after infection (Buonsenso et al., 2022). While these studies have contributed valuable information to coaches on conducting training safely, they have primarily focused on analyzing psychological performance after infection. As such, they have not tracked or described the changes that occur while returning to training.

It is also worth noting that many recommendations for athletes returning to training were made when the endemic strains worldwide were different from the current widespread Omicron strain. Previous research has relied on data from the original strain or other early strains that caused severe symptoms and data from hospitalized patients. Nonetheless, early studies found that the severity of post-infection sequelae is related to the severity of symptoms during infection, at least in terms of psychological aspects (Alzueta et al., 2022; Merikanto et al., 2022; Joli et al., 2022). Some studies suggest that Omicron-induced symptoms are relatively mild, with lower proportions of long COVID reported by patients infected with the Omicron strain than those infected with Beta or Delta strains (Arjun et al., 2023; Jassat et al., 2023). A study on student-athletes revealed that their antigen test results return to negative within 7 days after infection (Tsao et al., 2022), which implies that athletes infected with Omicron may have a more positive change in psychological factors after returning to training.

However, other studies have found that the prevalence of sleep disorders is higher in patients infected with Omicron than those infected with Delta (Sunada et al., 2022), and some researchers have noted no significant differences in specific symptoms, such as fatigue, between Omicron and Delta strains (Magnusson et al., 2022). Furthermore, an analysis of the symptoms of over 80,000 people infected with the Delta and Omicron strains found both at similar risk for cognitive and emotional impairment (Taquet et al., 2022). Changes in psychological factors in athletes who return to training may vary depending on the strain. Still, studies have yet to investigate this topic specifically to provide recommendations in the context of epidemic development following the widespread Omicron strain.

This study investigates changes in psychological factors, including fatigue, sleep, and mood, among athletes who return to training during the acute and subacute phases of recovery from infection after a widespread infection with the Omicron strain in athletes of Shanghai sports teams in December 2022. By conducting a series of measurements and analyses, this study intends to provide evidence-based recommendations for coaches and athletes regarding safe and effective strategies for returning to training after infection. The findings of this study may contribute to a better understanding of the recovery process of athletes after infection, as well as inform the development of effective management strategies for their safe return to training.

The study was divided into two parts. In the first part, the subjective experiences of athletes were analyzed using self-report scales. Given the aggregated nature of athlete infection and return to training, the study initially analyzed changes after different training durations by grouping athletes based on the time of their return to training. Later, the study examined changes in psychological factors again after a longer fixed time interval. In the second part, the characteristics of fatigue were supplemented by analyzing the characteristics of exercise-induced subjective fatigue changes in combination with an exercise task.

This research is a response to the recommendation of conducting psychological monitoring after returning to training, as suggested by previous studies (Wilson et al., 2020; Lindsay et al., 2021; Giusto & Asplund, 2022; McKinney et al., 2021; Elliott et al., 2022). To our knowledge, this is the first study to investigate such changes in athletes after an Omicron infection. It is worth noting that this study was carried out on a sample of Chinese athletes, and cultural influences were inevitable. While some studies have indicated that Chinese cultural characteristics may have the ability to mitigate the negative psychological effects of COVID-19 (Yap et al., 2021), there are also substantial similarities across cultures (Sugawara et al., 2022). The recommendations derived from this study can serve as a reference not only for Chinese athletes but also for athletes from other cultures and can be adapted in practice along with research on the impact of COVID-19 on the psychological state in local cultures.

## Materials and Methods

### Participants

A total of 267 athletes who resumed training in early January 2023 at the Chongming Sports Training Base in Shanghai participated in the first test. Among them, 31 athletes had not obtained a positive result in the nucleic acid or antigen test before the test (some athletes had suspected symptoms and had recovered), while six athletes reported having been infected before December 2022 due to personal and competition reasons and had already returned to regular training. After excluding these participants, a total of 230 athletes were included in the study, comprising 117 males and 113 females with a mean age of 18.62 years (*SD* = 4.16). These athletes participated in various sports, including fencing, modern pentathlon, boxing, judo, badminton, table tennis, gymnastics, martial arts, and handball.

One hundred and sixty-seven athletes participated in the second test after 1 week, with 86 males and 81 females (mean age 19.16 years, *SD* = 4.09). One hundred and twenty-four athletes participated in the third test after a further two-week interval, comprising 64 males and 60 females with a mean age of 18.74 years (*SD* = 3.87). Ninety-nine of these athletes completed the scale in all three tests, with 53 males and 46 females (mean age 18.99 years, *SD* = 3.93). Moreover, 117 athletes completed the exercise task in all three tests, consisting of 62 males and 55 females, with a mean age of 18.81 years (*SD* = 3.85).

The study protocol was approved by the Ethics Committee of the Shanghai Research Institute of Sports Science (Shanghai Anti-doping Agency) (Ethics Approval Number LLSC20230001), and complies with ethical principles stated in the latest version of the Declaration of Helsinki. All participants provided written consent before the first test.

### Design

**Figure 1:**
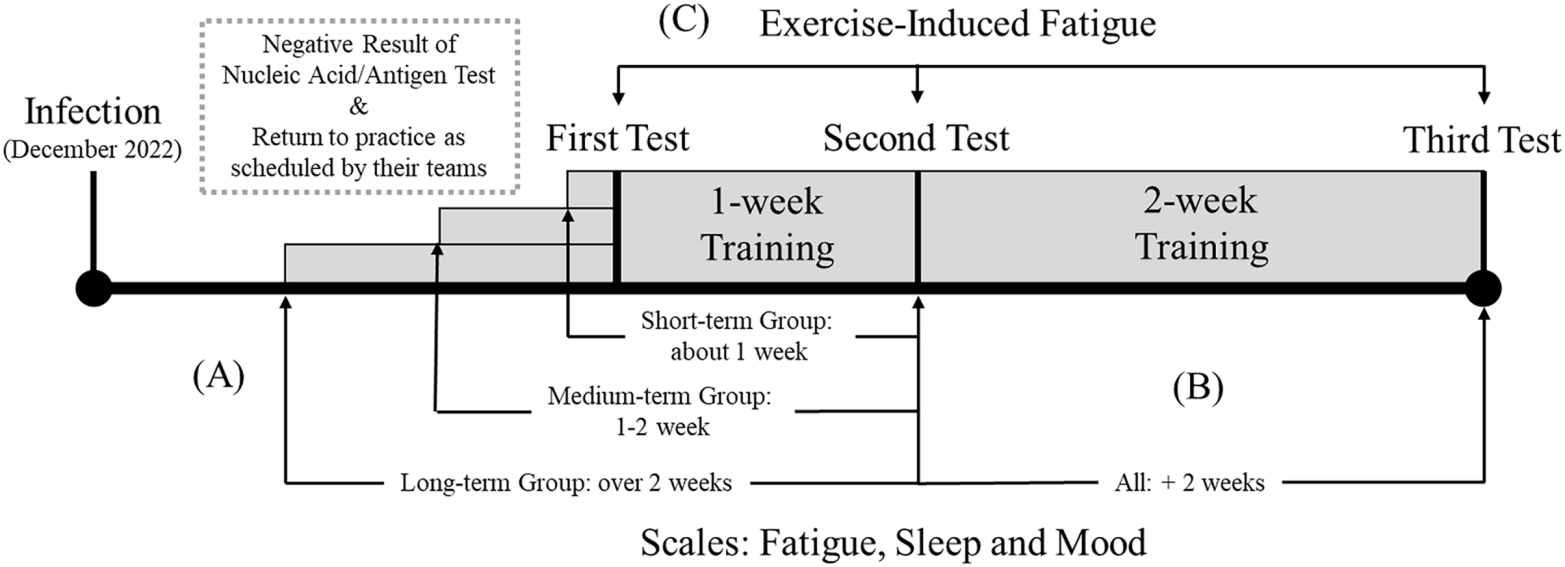
Overview of the procedure.

This study was structured into two main parts, utilizing quasi-experimental designs. The designs are described in Fig. 1 in association with the entire study procedure. The first part examines the impact of the duration of return to training on athletes’ fatigue, sleep, and mood, using self-report scales. Initially, athletes who returned to training in batches were divided into three groups based on the length of time they had been back in training. A 3 (duration: short, medium, and long) × 2 (test: pre-test, post-test) mixed design was employed, with time as a between-subjects variable and test point as a within-subjects variable. The pre-test refers to the athlete’s state when they first returned to training, and the post-test refers to the state at the second test, conducted after 1 week of training. By the time of the second test, the length of time the athlete had returned to training was classified into three categories: short (approximately 1 week), medium (1–2 weeks), and long (more than 2 weeks), as depicted in Fig. 1A. The dependent variables were the athletes’ mood, sleep, and fatigue status.

Additionally, the study analyzes the characteristics of psychological factors influenced by longer training periods. A one-factor two-level within-subjects design was employed to accomplish this, with the independent variable being the test (pre-test and post-test). The pre-test and post-test were the athletes’ mood, sleep, and fatigue status at the second and third tests, respectively, with approximately 2 weeks between tests, as shown in Fig. 1B.

The second part of the study analyzes the characteristics of exercise-induced subjective fatigue through an exercise task. A 3 (time: first test, second test, third test) × 2 (exercise: pre-exercise, post-exercise) within-subjects design was employed. The time refers to the three tests conducted, as depicted in Fig. 1C. The dependent variables were the level of physical and cognitive fatigue subjectively evaluated by the athletes.

### Procedure

From late December 2022 to early January 2023, most athletes from sports teams at the Shanghai Chongming Sports Training Base returned to the base to resume training. The criteria for resuming training were twofold: (1) the antigen/nucleic acid test must show a negative result after a period of rest in the dormitory or home isolation following infection, and (2) the athlete’s team can resume training and calls the athlete back to the training base. To ensure training safety, sports scientists at the training base conducted three sequential tests of athletic ability after the athletes’ return to training in early January. This study was conducted with these three tests (Fig. 1), each completed within 1 week.

Each test consisted of two parts: a test of fatigue, sleep, and mood scales and a test of exercise-induced fatigue. The athletes completed the scales on-site or 1 day in advance on a dedicated webpage. For the first test, athletes were asked to recall their status during the week when they returned to training. For the second and third tests, athletes were asked to respond based on their status during the previous week from the day of testing. Tests for exercise-induced fatigue were conducted both before and after exercise.

### Scales

(1) Symptoms questionnaire

The symptom questionnaire was developed based on the surveys used by medical institutions in Shanghai to investigate the symptoms (including fever, cough, sore throat, diarrhea, and other common symptoms) of the participants after infection and after they returned to training, as well as the dates of COVID-19-positive results in nucleic acid/antigen tests, their first negative results in nucleic acid/antigen tests after infection, and their return to training.

(2) Patient global impression of change (PGIC)

The PGIC was used to evaluate the subjective perception of symptom change after an intervention. This scale has only one item, a 7-point scale (ranging from one for much worse to seven for much improved) that subjects selected based on their physical condition compared to the pre-intervention state. The scale has been used in a study on the effects of exercise on facilitating recovery after infection with COVID-19 (Araújo et al., 2023). In the present study, during the first test, subjects were required to compare their current status with that before the infection and their current status with that when they had obtained their first negative result in the nucleic acid or antigen test after infection. During the second and third tests, participants were required to compare their current status with the previous test.

(3) Visual analog scale—training and daily fatigue (VAS-TDF)

Perceived training and daily fatigue were evaluated using the visual analog scale. Four questions were asked about physical fatigue in training, cognitive fatigue in training, overall physical fatigue in life, and overall cognitive fatigue in life. Participants responded using either their mobile phones or paper-based questionnaires. On the mobile phone webpage, subjects had to drag the slider on a line that fits the length of the screen of the phone to the position that matched their feelings, and the webpage converted the score automatically according to the dragging distance (0–100; 0 means no fatigue at all, 100 represents the extreme point of fatigue). This method has been used in studies of both physical and cognitive fatigue in different sports and is effective in evaluating fatigue (Coimbra et al., 2021; Gündoğdu et al., 2021). This method has also been used in studies to measure the mental state of Chinese people during the COVID-19 pandemic (Yan et al., 2021). Athletes used VAS-TDF to assess fatigue in training and daily life in the current study.

(4) Athens Insomnia Scale for non-clinical application (AIS-NCA)

The AIS-NCA was revised from the Athens Insomnia Scale and consisted of seven items, each scored on a scale of 1–5, with each option indicated by the text adapted to that item. All items are divided into two dimensions, sleep problems, and daytime functioning, and can be calculated for both subscales and the total scale score, with higher scores indicating more severe sleep quality problems (Sattler, Seddig & Zerbini, 2021). The scale has been used in studies of sleep related to COVID-19 and has demonstrated good utilization (Zerbini et al., 2022). The Chinese version of the questionnaire used by the participants consists of six items, constituting the two dimensions. The internal consistency coefficients were good in this study, with Cronbach’s α = 0.688 for the whole scale, Cronbach’s α = 0.636 for sleep problems, and Cronbach’s α = 0.761 for daytime functioning (based on the data of the first test).

(5) Depression, Anxiety, and Stress Scale (DASS-21)

The DASS-21 consists of twenty-one items scored from 0 to 3, divided into three dimensions of depression, anxiety, and stress, with higher scores, indicating more severe symptoms (Gong et al., 2010; Lovibond & Lovibond, 1995). Researchers have tested the reliability of this scale in athletes during the epidemic, demonstrating its validity in evaluating depression, anxiety, and stress in this population (Vaughan, Edwards & MacIntyre, 2020). The scale has good cross-cultural validity among Chinese adolescents (Mellor et al., 2014). The internal consistency coefficients were good in this study, with Cronbach’s α = 0.928 for the whole scale, Cronbach’s α = 0.575 for depression, Cronbach’s α = 0.767 for anxiety, and Cronbach’s α = 0.838 for stress (based on the data of first test).

(6) Visual analog scale—exercise-induced fatigue (VAS-EIF)

A visual analog scale (VAS) with 10 cm lines was employed to assess exercise-induced fatigue in this study. This method is relatively efficient in evaluating acute fatigue (Schlichta et al., 2022). The questionnaire comprised five questions related to physical fatigue, cognitive fatigue, sleepiness, decreased thinking clarity, and attention, all of which were completed on a paper-based form. For each question, a 10-cm line segment was provided, and participants indicated their level of fatigue by marking a vertical line on the line segment. The longer the length of the marked line, the higher the level of fatigue, except for the question related to attention, where the opposite was true. Sleepiness, inability to think clearly, and attention were complementary to central fatigue. Sleepiness, an experience where sleep is the primary pathway to recovery, was used to complement feelings related to fatigue but with differences in recovery (Mairesse et al., 2017). The ability to think and to pay attention were modified from the NASA-task load index interpretation of mental demand (Hart & Staveland, 1988). VAS-EEF was used to evaluate athletes’ perceived fatigue before and after the exercise task.

(7) Karolinska Sleepiness Scale (KSS)

The KSS was employed in the present study to evaluate the current subjective level of sleepiness or alertness. It is a widely used scale in driving, sleep deprivation, and shift work studies. The KSS consists of only one item scored from 1 to 9 (one indicating extreme alertness, and nine indicating excessive sleepiness). A corresponding text label accompanies each option, and subjects should select the one that best matches their feelings (Baulk, Reyner & Horne, 2001; Miley, Kecklund & Åkerstedt, 2016). KSS complemented the VAS-EEF to measure fatigue levels before and after the exercise task.

### Six-minute stair climb test

The exercise task employed in this study was a six-minute stair climb test. This test was based on the test utilized in a previous study (Da Costa et al., 2017). As a submaximal test, the intensity in this study was adjusted to correspond with the participating athletes’ physical form and physical function. The test was divided into two phases, each lasting 3 min. In the first phase, athletes climbed up and down a stair of dimensions 15 cm in height and 30 cm in width at a frequency of 120 beats per minute. Three minutes later, the athletes immediately proceeded to the second phase, where they climbed up and down a higher step stair (40 cm for male and 30 cm for female athletes) at the same rate.

This task was a component of the athletic ability monitoring program implemented following athletes’ return to training. Before and after the exercise task, athletes were required to wear sports physiology data collection equipment and complete several tests. The pre-test was administered using the scales before the exercise. At the same time, the post-test was carried out using the scale after the post-exercise sports physiology data collection (approximately 3 min later).

### Statistical analysis

The data were processed and analyzed using Excel 2016 and SPSS 26.0. Athletes who did not complete the required scales or exercise tasks were excluded from the statistical analysis. Depending on the design, this study employed repeated measures ANOVA, one-sample *t*-tests, and paired-sample *t*-tests to analyze the data. These statistical tests have been shown to have relatively good tolerance and robustness to the data, and the use of correction values can also contribute to the results in a beneficial way (Games, Keselman & Clinch, 1979; Poncet et al., 2016; Sullivan & D’Agostino, 1992; Zimmerman & Zumbo, 1992). To ensure the reliability of the results, appropriate correction values were used in the ANOVA based on the results of tests such as the sphericity test. Moreover, G*power 3.1.9.2 was used to calculate the minimum sample size required for reliable results. Specifically, the athletes’ infection and symptom characteristics were evaluated using descriptive statistical results. At the same time, their subjective physical condition changes (PGIC) were tested using one-sample *t*-tests (PGIC = 4 for no significant difference). The minimum sample size was calculated to be 54 (effect size d = 0.50, α = 0.05, 1−β = 0.95). The data from the scales in the first and second tests were analyzed using repeated measures ANOVA with Bonferroni correction for multiple comparisons (Fig. 1A). For those that did not satisfy the sphericity test hypothesis, the Greenhouse-Geisser correction was utilized. The minimum sample size was calculated to be 66 (effect size f = 0.25, α = 0.05, 1−β = 0.95, *r* = 0.50). The data from the scales in the second and third tests were analyzed using paired-samples *t*-tests (Fig. 1B). It was confirmed that there were significant correlations between the two tests before the statistics (*p*s < 0.001). The minimum sample size was calculated to be 54 (effect size dz = 0.50, α = 0.05, 1−β = 0.95). The data from the exercise-induced fatigue in the three tests were analyzed using repeated measures ANOVA (Fig. 1C). For those that did not satisfy the sphericity test hypothesis, the Greenhouse-Geisser correction was utilized. The presence of a non-integer number of degrees of freedom in the statistical results indicated that the *p*values were corrected. The minimum sample size was calculated to be 28 (effect size f = 0.25, α = 0.05, 1−β = 0.95, *r* = 0.50).

## Results

### Symptoms and recovery

Among the 230 athletes, 87.82% reported fever after infection. Of those with fever, 96.53% remembered and reported their maximum body temperature, with a mean value of 39.26 °C (*SD* = 0.84). Additionally, 90.43% of athletes experienced dry cough, 89.13% experienced fatigue, 73.04% experienced sore throat, 56.09% experienced decreased smell/taste, 43.48% experienced diarrhea, 80.87% experienced muscle aches, and 79.57% experienced sleepiness. The mean duration from the initial positive result of nucleic acid or antigen test to a negative retest was 6.77 days (*SD* = 3.29; *n* = 229). It took 6.91 days (*SD* = 4.62; *n* = 229) for athletes to resume training after regaining a negative result.

Most athletes experienced dry cough (65.65%) at the first test and fatigue (64.78%). Additionally, 24.78% experienced sore throat, 24.35% experienced reduced smell/taste, 21.30% experienced diarrhea, 52.61% experienced muscle soreness, and 53.91% experienced sleepiness.

Upon initial return to training, athletes perceived their condition to be worse than before infection (*M* = 3.38, *SD* = 1.46), *t*(207) = −6.128, *p* < 0.001, Cohen’s d = −0.425. However, they perceived their physical condition to be better than when the nucleic acid or antigen test had just turned negative (*M* = 4.52, *SD* = 1.29), *t*(207) = 5.864, *p* < 0.001, Cohen’s d = 0.407. At the second test, athletes perceived their physical condition to be better than at the previous test (*M* = 4.751, *SD* = 1.00), *t*(168) = 9.782, *p* < 0.001, Cohen’s d = 0.752. Similarly, at the third test, athletes perceived their physical condition to be better than at the previous test (*M* = 5.00, *SD* = 1.09), *t*(107) = 9.577, *p* < 0.001, Cohen’s d = 0.922.

### Changes after different durations of training

The results of the descriptive statistics are shown in Table 1. There was only a main effect of test on overall experienced physical fatigue, *F*(1,164) = 11.197, *p* = 0.001, η_p_^2^ = 0.063; fatigue was lower on the post-test. There was no significant main effect of duration, *F*(2,164) = 2.599, *p* = 0.077, η_p_^2^ = 0.031, and no significant interaction, *F*(2,164) = 0.552, *p* = 0.577, η_p_^2^ = 0.007. Additionally, there was no significant main effect of the test on overall experienced cognitive fatigue, *F*(1,164) = 0.806, *p* = 0.371, η_p_^2^ = 0.005. There was also no significant main effect of duration, *F*(2,164) = 0.321, *p* = 0.726, η_p_^2^ = 0.004, and no significant interaction, *F*(2,164) = 2.485, *p* = 0.086, η_p_^2^ = 0.029.

**Table 1:** Descriptive statistics of fatigue, sleep, and mood before and after restarting training for various durations (*M* ± *SD*).

		Short term		Medium term		Long term	
		Pre-test	Post-test	Pre-test	Post-test	Pre-test	Post-test
Fatigue	Overall physical fatigue	54.71 ± 24.06	48.58 ± 19.41	59.54 ± 23.51	56.57 ± 17.90	53.87 ± 17.90	47.31 ± 20.67
	Overall cognitive fatigue	47.92 ± 25.46	42.05 ± 19.59	46.90 ± 24.59	47.65 ± 23.15	43.84 ± 18.90	45.00 ± 19.06
	Physical fatigue in training	56.68 ± 24.78	50.83 ± 20.41	60.97 ± 21.67	58.14 ± 22.64	58.84 ± 18.09	50.31 ± 18.43
	Cognitive fatigue in training	47.17 ± 25.94	43.25 ± 21.70	46.57 ± 25.11	48.79 ± 23.47	46.20 ± 19.63	44.96 ± 19.68
Sleep	Sleep problems	8.56 ± 2.26	7.90 ± 2.39	7.90 ± 2.41	7.60 ± 2.54	8.18 ± 2.34	7.44 ± 2.28
	Daytime function	8.37 ± 1.69	7.81 ± 1.80	8.84 ± 1.73	8.43 ± 1.64	8.51 ± 1.39	7.98 ± 1.76
	Total	16.93 ± 3.27	15.71 ± 3.76	16.75 ± 3.18	16.03 ± 3.46	16.69 ± 3.35	15.42 ± 3.62
Mood	Depress	3.98 ± 3.96	3.11 ± 3.10	4.67 ± 3.72	4.00 ± 3.51	3.96 ± 2.58	3.64 ± 3.43
	Anxiety	4.14 ± 3.88	3.24 ± 2.83	5.25 ± 3.14	3.89 ± 3.09	4.22 ± 2.69	3.02 ± 3.47
	Stress	2.81 ± 3.50	2.39 ± 2.95	3.06 ± 2.95	2.89 ± 3.48	2.56 ± 2.21	2.31 ± 2.94

Regarding physical fatigue experienced during training, there was only a main effect of test, *F*(1,164) = 16.266, *p* < 0.001, η_p_^2^ = 0.090, with perceived fatigue being lower on the post-test. However, there was no significant main effect of duration, *F*(2,164) = 1.575, *p* = 0.271, η_p_^2^ = 0.016, and no significant interaction, *F*(2,164) = 1.317, *p* = 0.271, η_p_^2^ = 0.016. Furthermore, there was no significant main effect of test on cognitive fatigue experienced during training, *F*(1,164) = 0.402, *p* = 0.527, η_p_^2^ = 0.002. There was no significant main effect for duration, *F*(2,164) = 0.247, *p* = 0.781, η_p_^2^ = 0.003, and no significant interaction, *F*(2,164) = 1.482, *p* = 0.230, η_p_^2^ = 0.018.

Overall sleep quality showed a significant main effect of test, *F*(1,164) = 20.012, *p* < 0.001, η_p_^2^ = 0.109, indicating better sleep quality on the post-test. However, no significant main effect of duration was found, *F*(2,164) = 0.164, *p* = 0.849, η_p_^2^ = 0.002, and no significant interaction was observed, *F*(2,164) = 0.587, *p* = 0.557, η_p_^2^ = 0.007. Regarding the sleep problem dimension, a significant main effect of test was observed, *F*(1,164) = 11.560, *p* = 0.001, η_p_^2^ = 0.066. Sleep disturbance was lower in the post-test. However, no significant main effect of duration was observed, *F*(2,164) = 0.864, *p* = 0.423, η_p_^2^ = 0.010, and no significant interaction was found, *F*(2,164) = 0.677, *p* = 0.509, η_p_^2^ = 0.008. A significant main effect of test was observed for the daytime function dimension, *F*(1,164) = 16.190, *p* < 0.001, η_p_^2^ = 0.090. Specifically, daytime dysfunction was lower in the post-test. Nevertheless, no significant main effect of duration was found, *F*(2,164) = 2.159, *p* = 0.119, η_p_^2^ = 0.026, and no significant interaction was observed, *F*(2,164) = 0.145, *p* = 0.865, η_p_^2^ = 0.002.

Regarding depression, a significant main effect of test was observed, *F*(1,164) = 8.879, *p* = 0.003, η_p_^2^ = 0.051. Scores on the post-test were lower. However, no significant main effect of duration was observed, *F*(2,164) = 0.978, *p* = 0.378, η_p_^2^ = 0.007, and no significant interaction was observed, *F*(2,164) = 0.590, *p* = 0.556, η_p_^2^ = 0.007. On anxiety, a significant main effect of test was observed, *F*(1,164) = 27.981, *p* < 0.001, η_p_^2^ = 0.146. Scores on the post-test were lower. However, no significant main effect of duration was found, *F*(2,164) = 1.957, *p* = 0.145, η_p_^2^ = 0.023, and no significant interaction was observed, *F*(2,164) = 0.434, *p* = 0.649, η_p_^2^ = 0.005. Regarding stress, there was no significant main effect of test, *F*(1,164) = 2.187, *p* = 0.141, η_p_^2^ = 0.013. Additionally, no significant main effect of duration was found, *F*(2,164) = 0.538, *p* = 0.585, η_p_^2^ = 0.007, and no significant interaction was observed, *F*(2,164) = 0.167, *p* = 0.846, η_p_^2^ = 0.002.

### Changes after a longer fixed training period

The results of the descriptive statistics are shown in Table 2. After an additional 2 weeks of training, a statistically significant increase in overall physical fatigue was observed in athletes, *t*(98) = −2.822, *p* = 0.006, Cohen’s d = −0.284, while no significant change was detected in overall cognitive fatigue, *t*(98) = −1.211, *p* = 0.229, Cohen’s d = −0.122. As for training-related fatigue, a significant increase was found in physical fatigue, *t*(98) = −2.123, *p* = 0.036, Cohen’s d = −0.213, while cognitive fatigue remained unchanged, *t*(98) = −1.463, *p* = 0.147, Cohen’s d = −0.147.

**Table 2:** Descriptive statistics of the effects of completing two additional weeks of training on fatigue, sleep, and mood (*M* ± *SD*).

		Pre-test	Post-test
Fatigue	Overall physical fatigue	50.79 ± 21.21	55.81 ± 20.32
	Overall cognitive fatigue	44.97 ± 20.58	46.97 ± 19.42
	Physical fatigue in training	53.20 ± 21.31	57.18 ± 20.85
	Cognitive fatigue in training	46.05 ± 22.22	48.85 ± 19.63
Sleep	Sleep problems	7.22 ± 2.42	7.13 ± 2.44
	Daytime function	7.97 ± 1.75	7.69 ± 1.78
	Total score	15.19 ± 3.63	14.82 ± 3.71
Mood	Depress	3.55 ± 3.11	2.71 ± 3.00
	Anxiety	3.23 ± 2.75	2.86 ± 2.67
	Stress	2.28 ± 2.86	1.80 ± 2.54

No significant changes were observed in overall sleep quality, *t*(98) = 1.059, *p* = 0.292, Cohen’s d = 0.106, sleep problems, *t*(98) = 0.380, *p* = 0.705, Cohen’s d = 0.038, and daytime function, *t*(98) = 1.572, *p* = 0.119, Cohen’s d = 0.158.

A significant decrease was found in the level of depression, *t*(98) = 3.256, *p* = 0.002, Cohen’s d = 0.327, while the level of anxiety remained unchanged, *t*(98) = 1.689, *p* = 0.094, Cohen’s d = 0.170. Moreover, the stress decreased significantly, *t*(98) = 2.265, *p* = 0.026, Cohen’s d = 0.228.

### Changes in exercise task-induced fatigue

The results of the descriptive statistics are shown in Table 3. There was a significant interaction on subjectively experienced physical fatigue, *F*(2,228) = 4.830, *p* = 0.009, η_p_^2^ = 0.041. Simple effects analysis showed no significant difference between the pre-test and post-test in the first test (*p* = 0.122) and second test (*p* = 0.113), with only a higher level of physical fatigue in the post-test of the third test than in the pre-test (*p* = 0.021). A significant main effect of time was found, *F*(1.834,209.121) = 12.732, *p* < 0.001, η_p_^2^ = 0.100. Physical fatigue was higher on the first test than on the second test (*p* < 0.001) and third test (*p* < 0.001), but there was no difference between the second test and third test (*p* > 0.999). There was no significant main effect of exercise, *F*(1,114) = 1.606, *p* = 0.208, η_p_^2^ = 0.014.

**Table 3:** Descriptive statistics of fatigue induced by exercise task (*M* ± *SD*).

	First test		Second test		Third test	
	Pre-test	Post-test	Pre-test	Post-test	Pre-test	Post-test
Physical fatigue	57.00 ± 22.59	54.26 ± 24.22	46.09 ± 23.55	48.70 ± 23.71	45.46 ± 25.03	49.71 ± 24.08
Cognitive fatigue	43.31 ± 22.10	44.56 ± 24.34	38.90 ± 21.70	37.61 ± 23.23	36.85 ± 22.82	38.61 ± 23.75
Sleepiness	49.15 ± 22.78	39.91 ± 25.23	44.66 ± 22.93	39.07 ± 24.10	43.83 ± 24.16	39.60 ± 25.31
Unable to think clearly	40.90 ± 23.55	37.85 ± 23.54	33.10 ± 21.81	32.43 ± 22.27	34.64 ± 21.58	33.33 ± 23.23
Attention	55.58 ± 22.90	60.31 ± 20.57	59.84 ± 20.16	60.30 ± 21.13	58.67 ± 21.13	57.38 ± 23.58
KSS	6.30 ± 1.51	5.60 ± 1.85	5.77 ± 1.38	5.35 ± 1.55	5.60 ± 1.46	5.30 ± 1.56

Regarding the subjective experience of cognitive fatigue, there was a significant main effect of time, *F*(1.882,214.511) = 8.064, *p* = 0.001, η_p_^2^ = 0.066. Cognitive fatigue was higher for the first test than for the second test (*p* = 0.001) and third test (*p* = 0.005), but there was no significant difference between the second test and third test (*p* > 0.999). There was no significant main effect of exercise, *F*(1,114) = 0.202, *p* = 0.654, η_p_^2^ = 0.002; nor a significant interaction, *F*(2,228) = 1.296, *p* = 0.276, η_p_^2^ = 0.011.

There was a significant main effect of exercise on subjectively experienced sleepiness, *F*(1,114) = 20.027, *p* < 0.001, η_p_^2^ = 0.149. Sleepiness was lower on the post-test than on the pre-test. There was no significant main effect of time, *F*(1,114) = 1.482, *p* = 0.229, η_p_^2^ = 0.013. Similarly, no significant interaction was observed, *F*(2,228) = 2.464, *p* = 0.087, η_p_^2^ = 0.021.

A significant main effect of time was found on the subjective experience of being unable to think clearly, *F*(1.810,206.296) = 8.505, *p* < 0.001, η_p_^2^ = 0.069. The thinking was less clear on the first test than on the second test (*p* < 0.001) and third test (*p* = 0.017), but there was no significant difference between the second test and the third test (*p* > 0.999). There was no significant main effect for exercise, *F*(1,114) = 2.388, *p* = 0.125, η_p_^2^ = 0.021. There was no significant interaction, *F*(1.810,206.340) = 0.544, *p* = 0.564, η_p_^2^ = 0.005.

In addition, there was no significant main effect of time on the subjective experience of attention, *F*(2,226) = 0.789, *p* = 0.455, η_p_^2^ = 0.007. Similarly, there was no significant main effect of exercise, *F*(1,113) = 1.003, *p* = 0.319, η_p_^2^ = 0.009. Furthermore, no significant interaction was observed, *F*(2,226) = 2.529, *p* = 0.082, η_p_^2^ = 0.022.

Regarding sleepiness or lack of alertness measured by the KSS, an interaction between time and exercise was observed, *F*(1.789,202.194) = 4.121, *p* = 0.021, η_p_^2^ = 0.035. Simple effects analysis showed that, for the pre-test, the level of sleepiness was significantly higher on the first test compared to the second test (*p* < 0.001) and third test (*p* < 0.001). However, there was no significant difference between the second and third tests (*p* = 0.588). For the post-test, there was no significant difference between all three tests (*p*_1–2_ = 0.169, *p*_1–3_ = 0.188, *p*_2-3_ > 0.999). Furthermore, there was a significant main effect of time, *F*(1.870,211.277) = 10.370, *p* < 0.001, indicating that sleepiness was higher during the first test compared to the second test (*p* = 0.001) and the third test (*p* = 0.001). However, there was no significant difference between the second and third tests (*p* = 0.984). Additionally, there was a significant main effect of exercise, *F*(1,113) = 25.963, *p* < 0.001, η_p_^2^ = 0.187, indicating that sleepiness levels were higher during the pre-test than the post-test.

## Discussion

After widespread infection with the Omicron strain, athletes typically obtained negative nucleic acid or antigen test results within about 1 week and resumed training as scheduled. The time required to achieve negative test results is comparable to that previously reported for student-athletes (Tsao et al., 2022). While most athletes still exhibited multiple symptoms when returning to training, these symptoms gradually diminished. Therefore, resuming training may not have had a significant negative impact on the athletes’ physical health.

Fatigue is one of the most noticeable symptoms after COVID-19 infection (Stengel et al., 2021), and it poses a significant challenge to ensuring safety during training. This study showed that after resuming training, athletes experienced significantly lower physical fatigue overall and during training, whereas the level of cognitive fatigue did not change significantly. This change may have occurred soon after resuming training. However, as the training period was extended, the athletes’ physical fatigue levels increased, while their cognitive fatigue remained unchanged.

For the changes in physical fatigue, it is essential to consider that exercise may have contributed to both the decrease and the increase in fatigue levels. Exercise significantly reduces fatigue levels as a form of recovery after a viral infection (Joli et al., 2022; Jimeno-Almazán et al., 2022). Upon returning to training, it is possible that training may have played an intervening role in the post-infection fatigue experienced by the athletes, leading to a reduction in subjective physical fatigue. Additionally, improvements in lifestyle routines, dietary nutrition, and interpersonal communication after returning to the sports team may have indirectly accelerated positive changes in terms of mood and recovery related to the fatigue experience (Barker-Davies et al., 2020; D’Arcy et al., 2021; Romdhani et al., 2022). However, after a more extended training period, the training load may gradually return to normal levels, resulting in training-induced fatigue. Nevertheless, since the training load was not monitored in this study, whether the observed fatigue levels exceed normal levels at this stage is unclear. The contribution of infection-induced fatigue cannot be further analyzed.

The present study did not find significant changes in cognitive fatigue among athletes. Although athletes have made positive improvements in exercise, socialization, nutrition, and sleep after returning to training, which are important ways to recover from cognitive fatigue (D’Arcy et al., 2021), these improvements have not significantly reduced cognitive fatigue. This phenomenon may be attributed to the complexity of fatigue itself and athletes’ recognition of fatigue. Notably, symptoms such as tiredness and reduced spontaneous muscle activation closely related to physical performance are also associated with central fatigue. This type of fatigue is more likely to be treated as physical fatigue during training, which makes it difficult for athletes to distinguish the central and peripheral components of physical fatigue performance (Pageaux & Lepers, 2018; Staiano et al., 2023). Although sports demand high cognitive abilities, athletes are primarily concerned about physical fatigue. Their awareness of cognitive fatigue may not be as prominent as in other professions where cognitive abilities are the primary competencies contributing to job performance.

When combined with a specific exercise task, physical fatigue was found to show an overall significant decrease, followed by maintenance afterward. However, after a longer training period, post-exercise fatigue levels were significantly elevated. This result complements the changes in fatigue levels found in the athletes’ generalized subjective evaluation. Probably, athletes do not recover from fatigue at the desired level after longer and possibly more intense training, resulting in a higher likelihood of experiencing fatigue when completing simple exercise tasks.

Regarding cognitive fatigue, the study found that athletes experienced a decrease in subjectively perceived cognitive fatigue, which remained stable over time, while the clarity of thought increased in the short term and then also remained stable. This suggests that athletes can report and perceive changes in cognitive fatigue when combined with specific task states or specific to what cognitive fatigue entails and demonstrates that athletes can experience positive changes in cognitive fatigue levels after a short training period. These results are consistent with previous research highlighting the beneficial effects of physical exercise and environmental changes on brain health (Barker-Davies et al., 2020; D’Arcy et al., 2021; Romdhani et al., 2022), and complement the results of evaluations using scales alone.

For cognitive fatigue, the study also revealed that sleepiness decreased after training, which may be attributed to increased arousal levels due to exercise (Lambourne & Tomporowski, 2010). It was also observed that sleepiness was only higher on the first pre-test than on the subsequent pre-tests, with no differences found on the post-tests. This suggests that restarting training can improve feelings related to cognitive fatigue within a short duration, and that the elevated state achieved after exercise is relatively stable. This result may partially indicate the effect of exercise training on fatigue (D’Arcy et al., 2021). However, attention levels did not change significantly. Given that the impact of exercise on cognitive performance is dependent on factors such as exercise duration, type, and cognitive ability, it can be argued that the choice of exercise task used in the present study may have resulted in less impact on attention levels compared to thinking abilities (Lambourne & Tomporowski, 2010).

Athletes experienced a significant improvement in sleep quality after resuming training, which occurred quickly after return but not again after a longer training period. This was observed in both sleep problems during the night and daytime functioning after waking. This improvement could be related to the change in the living environment after restarting training. Studies have demonstrated the significant impact of home isolation on sleep patterns (Gupta et al., 2020; Yuksel et al., 2021), and researchers have suggested interventions for sleep problems during home isolation management, such as outdoor activities, dietary management, and rest management (Romdhani et al., 2022), which are precisely the types of interventions that occur when returning to the daily management of a sports team after resuming training. Although there is no direct evidence, it can be inferred that athletes improved their sleep quality quickly after returning to training through a more scientific and disciplined daily routine. This improvement occurred quickly and did not show further differences across training times or over longer training periods. However, it is essential to note that athletes are at high risk for sleep quality problems (Gupta, Morgan & Gilchrist, 2017), and the lack of change in sleep quality after a longer training period does not necessarily indicate that athletes already have good sleep quality.

In terms of mood, athletes exhibited positive changes in various moods upon resuming training but showed different characteristics of changes depending on the specific mood. Depression showed a swift positive change soon after returning to training. It can be inferred that restarting training alone can help reduce depression levels. Additionally, extending the duration of training can further decrease depression levels. The returning may be responsible for this effect, resulting from two aspects. The first aspect is the very act of returning to training itself, which may contribute to the rapid improvement of depression after resuming training. Apart from the illness, the cessation of training and home isolation can also induce depressive experiences in athletes (Batalla-Gavalda et al., 2021). In a similar approach, rest in isolation after infection may additionally generate a certain level of depression. Resuming training may quickly alleviate overall depressed mood by eliminating the additional depression caused by home or dormitory isolation. This direct effect does not vary across recovery training durations (Mehrsafar et al., 2021; Rehman, Yıldırım & Shahnawaz, 2022).

The second aspect is exercise, which may explain the ability to decrease depression levels after longer training periods further. As training extends, the strength of exercise training gradually approaches normal levels and is maintained based on physical performance monitoring and the principle of gradual progression. Regular exercise is an effective way to intervene in depression (Kim, 2022). The fact that athletes train regularly for a more extended period at an appropriate intensity makes training itself an intervention tool.

Unlike depression, anxiety levels do not change quickly after the resumption of training but may only begin to decrease as training is extended further. Concerns about symptoms and individual performance may underlie this phenomenon. Some researchers have suggested that anxiety is a more common symptom than depression in chronic fatigue following viral infection, which is often related to concerns about one’s ability to achieve a desired state of performance (Gaber, 2021). Moreover, anxiety may also be associated with the stigmatization of the after-effects, which may be accentuated in an environment where social media messages are prevalent (Chasco et al., 2022). For athletes who seek to break their limits and set high personal goals, performance uncertainty may be a significant cause of anxiety. These causes may relate to the inability to judge whether competence has been recovered due to lower-than-normal protocols in the early stages of recovery training and to the knowledge of after-effects acquired through various sources. As the training load gradually returns to its usual level after an extended period of training and after a longer period of personal experience, the factors that trigger anxiety might gradually be eliminated, allowing the level of anxiety to decrease.

Stress differs from anxiety in terms of its changes. Stress can decrease after the return to training and is independent of the duration, but the change does not develop further as the duration of training is prolonged. It has been noted that depression, anxiety, and stress levels decrease substantially after release from isolation (Rehman, Yıldırım & Shahnawaz, 2022). Athletes who are released from home isolation and return to training may be positively affected by the discharge from isolation, resulting in reduced stress levels. The effects of release from isolation are accomplished in the brief term, so this effect is not reflected in more extended periods of training. The phenomenon of change in stress may also complement changes in anxiety. Stress is a constant state of arousal and tension that is more closely associated with anxiety and has symptomatic continuity with anxiety (Lovibond & Lovibond, 1995). A decrease in stress levels upon return to training may be a prerequisite for anxiety to decrease after a more extended training period.

This study represents the first attempt to track and analyze changes in psychological factors such as fatigue, sleep, and mood in athletes infected with omicron strains after returning to training as a response to return-to-training recommendations (Elliott et al., 2020; McKinney et al., 2021), and to provide psychological suggestions for return to training accordingly. The results of this study indicate that fatigue, sleep, and mood can be significantly improved in athletes after returning to training. Returning to training earlier after an infection (must after obtaining a negative result of nucleic acid or antigen test) not only promotes the maintenance of athletic ability but also has a positive effect on the recovery of psychological well-being. To further enhance the efficacy of returning to training, an assessment of psychological factors, including fatigue, sleep, and mood, should be conducted as soon as possible after returning to training and retested several times within at least 1 month. Additionally, emphasis on assessment and intervention for anxiety is required when athletes return to training, and monitoring of exercise fatigue and interventions for fatigue recovery should be reinforced after training. When considering the influence of possible cultural differences, the above recommendations need to be appropriately adapted in practical application, considering the results of research on the effects of COVID-19 on the psychological state in the local culture.

As for the limitations of the experimental conditions, it should be noted that the mass infection in athletes was an unexpected event, and there was a limited time window in the training program to conduct testing in this study. As a result, this study had to focus on the acute and subacute phases of the infection, which did not involve the “long COVID”. It lacked baseline and objectively measured data to determine recovery. Consequently, the scope of interpretation of the results is limited.

Another limitation of this study is that the same exercise task was used for athletes from different sports. While this study examined the changing characteristics of exercise-induced fatigue in combination with the exercise task, using such a uniform task may mask possible differences in sports, given that sports with different aerobic/anaerobic capacity demands may differ in post-infection fatigue (Buonsenso et al., 2022). Future studies should consider more refined recovery recommendations for athlete fatigue in conjunction with sport-specific training tasks, especially in cases of sporadic infection occurrence.

Finally, cultural considerations should also be taken into account. This study focused only on the athlete population in Chinese culture, so it is premature to generalize the findings and recommendations to other cultures or populations. Although there are cross-cultural commonalities in mental health changes and factors influencing mental health during epidemics, inter-cultural differences also exist (Sugawara et al., 2022; Yap et al., 2021) and require further research. Moreover, the cross-cultural properties of the measurement instruments used in the study in China or the cross-population characteristics in the population of athletes need to be further investigated (Mellor et al., 2014; Oei et al., 2013), which may complicate cross-cultural comparisons and experience summaries. Thus, future research should emphasize cross-cultural comparisons and updating measurement instruments to facilitate the summary of studies and the exploration of cross-cultural phenomena.

## Conclusions

This study provides preliminary evidence of the positive effects of returning to training after infection on the psychological status of athletes during the period when the Omicron strain was prevalent. Although returning to training has a positive effect on the emotional, sleep, and fatigue status of athletes with quick results, it is still necessary to regularly assess their psychological status just after returning to training. Attention should be given to enhancing assessment and interventions for anxiety in the short term after return and for fatigue after a period of training. Following these recommendations, it is hoped that athletes infected with the Omicron strain can safely and efficiently return to training.

## Supplemental Information

10.7717/peerj.15580/supp-1 Supplemental Information 1 Data of scales.

10.7717/peerj.15580/supp-2 Supplemental Information 2 Data: Exercise induced fatigue.

10.7717/peerj.15580/supp-3 Supplemental Information 3 Symptoms questionnaire: Chinese.

10.7717/peerj.15580/supp-4 Supplemental Information 4 Symptoms questionnaire: Translated.

## Additional Information and Declarations

### Data Availability

The following information was supplied regarding data availability: The raw measurements are available in the Supplemental Files.

## References

[ref-1] Abd-Elfattah HM, Abdelazeim FH, Elshennawy S (2015). Physical and cognitive consequences of fatigue: a review. Journal of Advanced Research.

[ref-2] Almonroeder TG, Tighe SM, Miller TM, Lanning CR (2020). The influence of fatigue on decision-making in athletes: a systematic review. Sports Biomechanics.

[ref-3] Alzueta E, Perrin PB, Yuksel D, Ramos-Usuga D, Kiss O, Iacovides S, de Zambotti M, Cortes M, Olabarrieta-Landa L, Arango-Lasprilla JC (2022). An international study of post-COVID sleep health. Sleep Health.

[ref-4] Araújo BTS, Barros AEVR, Nunes DTX, Remígio De Aguiar MI, Mastroianni VW, de Souza JAF, Fernades J, Campos SL, Brandão DC, Dornelas De Andrade A (2023). Effects of continuous aerobic training associated with resistance training on maximal and submaximal exercise tolerance, fatigue, and quality of life of patients post-COVID-19. Physiotherapy Research International.

[ref-5] Arjun MC, Singh AK, Roy P, Ravichandran M, Mandal S, Pal D, Das K, Gajjala A, Venkateshan M, Mishra B, Patro BK, Mohapatra PR, Subba SH (2023). Long COVID following Omicron wave in Eastern India—a retrospective cohort study. Journal of Medical Virology.

[ref-6] Barker-Davies RM, O’Sullivan O, Senaratne KPP, Baker P, Cranley M, Dharm-Datta S, Ellis H, Goodall D, Gough M, Lewis S (2020). The Stanford Hall consensus statement for post-COVID-19 rehabilitation. British Journal of Sports Medicine.

[ref-7] Batalla-Gavalda A, Cecilia-Gallego P, Revillas-Ortega F, Beltran-Garrido JV (2021). Variations in the mood states during the different phases of COVID-19’s lockdown in young athletes. International Journal of Environmental Research and Public Health.

[ref-8] Baulk SD, Reyner LA, Horne JA (2001). Driver sleepiness—evaluation of reaction time measurement as a secondary task. Sleep.

[ref-9] Buonsenso A, Murri A, Centorbi M, Di Martino G, Calcagno G, di Cagno A, Fiorilli G, Iuliano E (2022). Psychological wellbeing and perceived fatigue in competitive athletes after SARS-CoV-2 infection 2 years after pandemic start: practical indications. Journal of Functional Morphology and Kinesiology.

[ref-10] Chasco EE, Dukes K, Jones D, Comellas AP, Hoffman RM, Garg A (2022). Brain fog and fatigue following COVID-19 infection: an exploratory study of patient experiences of long COVID. International Journal of Environmental Research and Public Health.

[ref-11] Coimbra DR, Bevilacqua GG, Pereira FS, Andrade A (2021). Effect of mindfulness training on fatigue and recovery in elite volleyball athletes: a randomized controlled follow-up study. Journal of Sports Science & Medicine.

[ref-12] Corona VF, Gualano MR, Rossi MF, Valz Gris A, Amantea C, Moscato U, Ricciardi W (2022). Psychological and mental sequelae in elite athletes with previous SARS-CoV-2 infection: a systematic review. International Journal of Environmental Research and Public Health.

[ref-13] Da Costa CH, Da Silva KM, Maiworm A, Raphael Y, Parnayba J, Da Cal M, Figueira B, Condesso D, Rufino R (2017). Can we use the 6-minute step test instead of the 6-minute walking test? An observational study. Physiotherapy.

[ref-14] D’Arcy RC, Sandhu JK, Marshall S, Besemann M (2021). Mitigating long-term COVID-19 consequences on brain health. Frontiers in Neurology.

[ref-15] Elliott N, Biswas A, Heron N, Ranson C, Hull J, Martin R, Elliott J (2022). Graduated Return to Play after SARS-CoV-2 infection – what have we learned and why we’ve updated the guidance. British Journal of Sport Medicine (BJSM).

[ref-16] Elliott N, Martin R, Heron N, Elliott J, Grimstead D, Biswas A (2020). Infographic. Graduated return to play guidance following COVID-19 infection. British Journal of Sports Medicine.

[ref-17] Fiorilli G, Buonsenso A, Davola N, Di Martino G, Baralla F, Boutious S, Centorbi M, Calcagno G, di Cagno A (2021). Stress impact of COVID-19 sports restrictions on disabled athletes. International Journal of Environmental Research and Public Health.

[ref-18] Gaber T (2021). Assessment and management of post-COVID fatigue. Progress in Neurology and Psychiatry.

[ref-19] Games PA, Keselman HJ, Clinch JJ (1979). Tests for homogeneity of variance in factorial designs. Psychological Bulletin.

[ref-20] Giusto E, Asplund CA (2022). Persistent COVID and a return to sport. Current Sports Medicine Reports.

[ref-21] Goldberg EE, Lin Q, Romero-Severson EO, Ke R (2023). Quantifying the rate and magnitude of the Omicron outbreak in China after sudden exit from ‘zero-COVID’ restrictions. medRxiv.

[ref-22] Gong X, Xie X, Xu R, Luo Y (2010). Psychometric properties of the chinese versions of DASS-21 in Chinese college students. Chinese Journal of Clinical Psychology.

[ref-25] Gündoğdu S, Çolak ÖH, Doğan EA, Gülbetekin E, Polat Ö (2021). Assessment of mental fatigue and stress on electronic sport players with data fusion. Medical & Biological Engineering & Computing.

[ref-23] Gupta R, Grover S, Basu A, Krishnan V, Tripathi A, Subramanyam A, Nischal A, Hussain A, Mehra A, Ambekar A (2020). Changes in sleep pattern and sleep quality during COVID-19 lockdown. Indian Journal of Psychiatry.

[ref-24] Gupta L, Morgan K, Gilchrist S (2017). Does elite sport degrade sleep quality? A systematic review. Sports Medicine.

[ref-26] Halson SL (2013). Recovery techniques for athletes. Sports Science Exchange.

[ref-27] Hart SG, Staveland LE (1988). Development of NASA-TLX (Task Load Index): results of empirical and theoretical research. Advances in Psychology.

[ref-28] Huang J, Zhao S, Chong KC, Zhou Y, Lu W, Fang F, Cheung PPH, Lai KC, Hui DS, Mok CKP (2023). Infection rate in Guangzhou after easing the zero-COVID policy: seroprevalence results to ORF8 antigen. The Lancet Infectious Diseases.

[ref-29] Hughes DC, Orchard JW, Partridge EM, La Gerche A, Broderick C (2022). Return to exercise post-COVID-19 infection: a pragmatic approach in mid-2022. Journal of Science and Medicine in Sport.

[ref-30] Jassat W, Mudara C, Vika C, Welch R, Arendse T, Dryden M, Blumberg L, Mayet N, Tempia S, Parker A, Nel J, Perumal R, Groome MJ, Conradie F, Ndjeka N, Sigfrid L, Merson L, Cohen C (2023). A cohort study of post-COVID-19 condition across the Beta, Delta, and Omicron waves in South Africa: 6-month follow-up of hospitalized and nonhospitalized participants. International Journal of Infectious Diseases.

[ref-31] Jimeno-Almazán A, Franco-López F, Buendía-Romero Á, Martínez-Cava A, Sánchez-Agar JA, Sánchez-Alcaraz Martínez BJ, Courel-Ibáñez J, Pallarés JG (2022). Rehabilitation for post-COVID-19 condition through a supervised exercise intervention: a randomized controlled trial. Scandinavian Journal of Medicine & Science in Sports.

[ref-32] Joli J, Buck P, Zipfel S, Stengel A (2022). Post-COVID-19 fatigue: a systematic review. Frontiers in Psychiatry.

[ref-33] Kim J (2022). Regular physical exercise and its association with depression: a population-based study short title: exercise and depression. Psychiatry Research.

[ref-34] Kölling S, Duffield R, Erlacher D, Venter R, Halson SL (2019). Sleep-related issues for recovery and performance in athletes. International Journal of Sports Physiology and Performance.

[ref-35] Lambourne K, Tomporowski P (2010). The effect of exercise-induced arousal on cognitive task performance: a meta-regression analysis. Brain Research.

[ref-36] Leung K, Lau EHY, Wong CKH, Leung GM, Wu JT (2023). Estimating the transmission dynamics of SARS-CoV-2 Omicron BF.7 in Beijing after adjustment of the zero-COVID policy in November–December 2022. Nature Medicine.

[ref-37] Liang J, Liu R, He W, Zeng Z, Wang Y, Wang B, Liang L, Zhang T, Chen CLP, Chang C, Hon C, Lau EHY, Yang Z, Tong K (2023). Infection rates of 70% of the population observed within 3 weeks after release of COVID-19 restrictions in Macao, China. Journal of Infection.

[ref-38] Lima Y, Denerel N, Devran S, Rice S, Bayraktar B (2022). Which athletes are more vulnerable to mental health symptoms during the COVID-19 crisis? A cross-sectional study. Research in Sports Medicine.

[ref-39] Lima Y, Denerel N, Öz ND, Senisik S (2021). The psychological impact of COVID-19 infection on athletes: example of professional male football players. Science and Medicine in Football.

[ref-40] Lindsay RK, Wilson JJ, Trott M, Olanrewaju O, Tully MA, López-Sánchez GF, Shin JI, Pizzol D, Allen P, Butler LT, Barnett Y, Smith L (2021). What are the recommendations for returning athletes who have experienced long term COVID-19 symptoms?. Annals of Medicine.

[ref-41] Lovibond PF, Lovibond SH (1995). The structure of negative emotional states: comparison of the Depression Anxiety Stress Scales (DASS) with the beck depression and anxiety inventories. Behaviour Research and Therapy.

[ref-42] Magnusson K, Kristoffersen DT, Dell Isola A, Kiadaliri A, Turkiewicz A, Runhaar J, Bierma-Zeinstra S, Englund M, Magnus PM, Kinge JM (2022). Post-covid medical complaints following infection with SARS-CoV-2 Omicron vs Delta variants. Nature Communications.

[ref-43] Mairesse O, Damen V, Newell J, Kornreich C, Verbanck P, Neu D (2017). The Brugmann fatigue scale: an analogue to the epworth sleepiness scale to measure behavioral rest propensity. Behavioral Sleep Medicine.

[ref-44] Malhotra RK (2017). Sleep, recovery, and performance in sports. Neurologic Clinics.

[ref-45] Manu P (2022). Post-COVID fatigue: functional brain imaging and cognitive testing. American Journal of Therapeutics.

[ref-46] McKinney J, Connelly KA, Dorian P, Fournier A, Goodman JM, Grubic N, Isserow S, Moulson N, Philippon F, Pipe A (2021). COVID-19-myocarditis and return to play: reflections and recommendations from a Canadian working group. Canadian Journal of Cardiology.

[ref-47] Mehrsafar AH, Moghadamzadeh A, Gazerani P, Jaenes Sanchez JC, Nejat M, Rajabian Tabesh M, Abolhasani M (2021). Mental health status, life satisfaction, and mood state of elite athletes during COVID 19 pandemic: a follow-up study in the phases of home confinement, reopening, and semi-lockdown condition. Frontiers in Psychology.

[ref-48] Mellor D, Vinet EV, Xu X, Hidayah Bt Mamat N, Richardson B, Román F (2014). Factorial invariance of the DASS-21 among adolescents in four countries. European Journal of Psychological Assessment.

[ref-49] Merikanto I, Dauvilliers Y, Chung F, Wing YK, De Gennaro L, Holzinger B, Bjorvatn B, Morin CM, Penzel T, Benedict C (2022). Sleep symptoms are essential features of long-COVID-comparing healthy controls with COVID-19 cases of different severity in the international COVID sleep study (ICOSS-II). Journal of Sleep Research.

[ref-50] Miley AÅ, Kecklund G, Åkerstedt T (2016). Comparing two versions of the Karolinska Sleepiness Scale (KSS). Sleep and Biological Rhythms.

[ref-51] Mon-López D, de la Rubia Riaza A, Hontoria Galán M, Refoyo Roman I (2020). The impact of Covid-19 and the effect of psychological factors on training conditions of handball players. International Journal of Environmental Research and Public Health.

[ref-52] Nowakowski S, Kokonda M, Sultana R, Duong BB, Nagy SE, Zaidan MF, Baig MM, Grigg BV, Seashore J, Deer RR (2022). Association between sleep quality and mental health among patients at a post-COVID-19 recovery clinic. Brain Sciences.

[ref-53] Oei TP, Sawang S, Goh YW, Mukhtar F (2013). Using the depression anxiety stress scale 21 (DASS-21) across cultures. International Journal of Psychology.

[ref-54] O’Donnell S, Beaven CM, Driller MW (2018). From pillow to podium: a review on understanding sleep for elite athletes. Nature and Science of Sleep.

[ref-55] Pageaux B, Lepers R (2018). The effects of mental fatigue on sport-related performance. Progress in Brain Research.

[ref-56] Phelan D, Kim JH, Elliott MD, Wasfy MM, Cremer P, Johri AM, Emery MS, Sengupta PP, Sharma S, Martinez MW (2020). Screening of potential cardiac involvement in competitive athletes recovering from COVID-19: an expert consensus statement. Cardiovascular Imaging.

[ref-57] Poncet A, Courvoisier DS, Combescure C, Perneger TV (2016). Normality and sample size do not matter for the selection of an appropriate statistical test for two-group comparisons. Methodology-European Journal of Research Methods for the Behavioral and Social Sciences.

[ref-58] Rehman U, Yıldırım M, Shahnawaz MG (2022). A longitudinal study of depression, anxiety, and stress among Indians during COVID-19 pandemic. Psychology, Health & Medicine.

[ref-59] Roelands B, Kelly V, Russell S, Habay J (2021). The physiological nature of mental fatigue: current knowledge and future avenues for sport science. International Journal of Sports Physiology and Performance.

[ref-60] Romdhani M, Rae DE, Nédélec M, Ammar A, Chtourou H, Al Horani R, Ben Saad H, Bragazzi N, Dönmez G, Driss T (2022). COVID-19 lockdowns: a worldwide survey of circadian rhythms and sleep quality in 3911 athletes from 49 countries, with data-driven recommendations. Sports Medicine.

[ref-61] Sattler S, Seddig D, Zerbini G (2021). Assessing sleep problems and daytime functioning: a translation, adaption, and validation of the Athens Insomnia Scale for non-clinical application (AIS-NCA).

[ref-62] Scarpelli S, De Santis A, Alfonsi V, Gorgoni M, Morin CM, Espie C, Merikanto I, Chung F, Penzel T, Bjorvatn B, Dauvilliers Y, Holzinger B, Wing YK, Partinen M, Plazzi G, De Gennaro L (2022). The role of sleep and dreams in long-COVID. Journal of Sleep Research.

[ref-63] Schlichta C, Cabral LL, Da Silva CK, Bigliassi M, Pereira G (2022). Exploring the impact of mental fatigue and emotional suppression on the performance of high-intensity endurance exercise. Perceptual and Motor Skills.

[ref-64] Shen X, Wang P, Shen J, Jiang Y, Wu L, Nie X, Liu J, Chen W (2023). Neurological manifestations of hospitalized patients with mild to moderate infection with SARS-CoV-2 Omicron variant in Shanghai. China Journal of Infection and Public Health.

[ref-65] Staiano W, Bonet LRS, Romagnoli M, Ring C (2023). Mental fatigue: the cost of cognitive loading on weight lifting, resistance training, and cycling performance. International Journal of Sports Physiology and Performance.

[ref-66] Stengel A, Malek N, Zipfel S, Goepel S (2021). Long haulers—what is the evidence for post-COVID fatigue?. Frontiers in Psychiatry.

[ref-67] Su Z, McDonnell D, Cheshmehzangi A, Ahmad J, Šegalo S, Pereira Da Veiga C, Xiang Y (2022). How to join and stay in the Olympic COVID-free bubble?. Brain, Behavior, & Immunity—Health.

[ref-68] Sugawara D, Chishima Y, Kubo T, Shah RIAB, Phoo EYM, Ng SL, Masuyama A, Gu Y, Tee EYJ (2022). Mental health and psychological resilience during the COVID-19 pandemic: a cross-cultural comparison of Japan, Malaysia, China, and the U.S. Journal of Affective Disorders.

[ref-69] Sullivan LM, D’Agostino RB (1992). Robustness of the t test applied to data distorted from normality by floor effects. Journal of Dental Research.

[ref-70] Sunada N, Nakano Y, Otsuka Y, Tokumasu K, Honda H, Sakurada Y, Matsuda Y, Hasegawa T, Omura D, Ochi K, Hagiya H, Ueda K, Kataoka H, Otsuka F (2022). Characteristics of sleep disturbance in patients with long COVID: a retrospective observational study in Japan. Journal of Clinical Medicine.

[ref-71] Taquet M, Sillett R, Zhu L, Mendel J, Camplisson I, Dercon Q, Harrison PJ (2022). Neurological and psychiatric risk trajectories after SARS-CoV-2 infection: an analysis of 2-year retrospective cohort studies including 1 284 437 patients. The Lancet Psychiatry.

[ref-72] Toresdahl BG, Asif IM (2020). Coronavirus disease 2019 (COVID-19): considerations for the competitive athlete. Sports Health-A Multidisciplinary Approach.

[ref-73] Tsao J, Kussman A, Segovia NA, Abrams GD, Boehm AB, Hwang CE (2022). Prevalence of positive rapid antigen tests after 7-day isolation following SARS-CoV-2 infection in college athletes during Omicron variant predominance. JAMA Network Open.

[ref-84] Üngür G, Karagözoğlu C (2021). Do personality traits have an impact on anxiety levels of athletes during the COVID-19 pandemic?. Current Issues in Personality Psychology.

[ref-74] Vaughan RS, Edwards EJ, MacIntyre TE (2020). Mental health measurement in a post Covid-19 world: psychometric properties and invariance of the DASS-21 in athletes and non-athletes. Frontiers in Psychology.

[ref-75] Washif JA, Kok L, James C, Beaven CM, Farooq A, Pyne DB, Chamari K (2023). Athlete level, sport-type, and gender influences on training, mental health, and sleep during the early COVID-19 lockdown in Malaysia. Frontiers in Physiology.

[ref-76] Washif JA, Mohd Kassim SFA, Lew PCF, Chong CSM, James C (2021). Athlete’s perceptions of a quarantine training camp during the COVID-19 lockdown. Frontiers in Sports and Active Living.

[ref-77] Wilson MG, Hull JH, Rogers J, Pollock N, Dodd M, Haines J, Harris S, Loosemore M, Malhotra A, Pieles G (2020). Cardiorespiratory considerations for return-to-play in elite athletes after COVID-19 infection: a practical guide for sport and exercise medicine physicians. British Journal of Sports Medicine.

[ref-78] Yan S, Xu R, Stratton TD, Kavcic V, Luo D, Hou F, Bi F, Jiao R, Song K, Jiang Y (2021). Sex differences and psychological stress: responses to the COVID-19 pandemic in China. BMC Public Health.

[ref-79] Yap S, Lee A, Ji L, Li Y, Dong Y (2021). Cultural differences in people’ s psychological response to COVID-19. Frontiers in Psychology.

[ref-80] Yuksel D, McKee GB, Perrin PB, Alzueta E, Caffarra S, Ramos-Usuga D, Arango-Lasprilla JC, Baker FC (2021). Sleeping when the world locks down: correlates of sleep health during the COVID-19 pandemic across 59 countries. Sleep Health.

[ref-81] Zerbini G, Taflinger S, Reicherts P, Kunz M, Sattler S (2022). Perceived risk of COVID-19 exposure and poor COVID-19 prognosis impair sleep: the mediating and moderating roles of COVID-19-related anxiety and knowledge. Journal of Sleep Research.

[ref-82] Zhou Y, Zhi H, Teng Y (2023). The outbreak of SARS-CoV-2 Omicron lineages, immune escape, and vaccine effectivity. Journal of Medical Virology.

[ref-83] Zimmerman DW, Zumbo BD (1992). Parametric alternatives to the student T test under violation of normality and homogeneity of variance. Perceptual and Motor Skills.

