# Supplementary material for: Psychological changes in athletes infected with Omicron after return to training: fatigue, sleep, and mood
Source: PeerJ. 2023 Jun 15;11:e15580. doi: 10.7717/peerj.15580 (PMC10276985; doi:10.7717/peerj.15580)
Supplement: Supplemental Information 3 [file peerj-11-15580-s003.docx]

**新冠病毒感染后症状问卷（第1次测试）**

姓名：_____ 运动队：______

- 首次抗原或核酸结果阳性日期：______________
- 阳性后，抗原或核酸首次显示阴性日期：______________
- 恢复训练的日期：______________
- 感染期间是否发烧？ 是 否
- 上一题选择“是”，请回答发烧的最高温度：__________
- 感染期间是否有以下症状？（勾选）

| 干咳 |  |
| --- | --- |
| 乏力 |  |
| 咽痛 |  |
| 嗅/味觉减退 |  |
| 腹泻 |  |
| 肌肉酸痛 |  |
| 嗜睡 |  |

- 截止现在是否还有如下症状？（勾选）

| 干咳 |  |
| --- | --- |
| 乏力 |  |
| 咽痛 |  |
| 嗅/味觉减退 |  |
| 腹泻 |  |
| 肌肉酸痛 |  |
| 嗜睡 |  |

- 相比感染病毒（测出阳性前）的时候，你感到自己现在的状态：

（ ）变得非常糟了

（ ）变糟了

（ ）有些变糟了

（ ）没变化

（ ）有些改善了

（ ）改善了

（ ）有了非常大的改善

- 相比刚刚转为阴性的时候，你感到自己现在的状态：

（ ）变得非常糟了

（ ）变糟了

（ ）有些变糟了

（ ）没变化

（ ）有些改善了

（ ）改善了

（ ）有了非常大的改善

**新冠病毒感染后症状问卷（第2/3次测试）**

姓名：_____ 运动队：______

- 截止现在是否还有如下症状？（勾选）

| 干咳 |  |
| --- | --- |
| 乏力 |  |
| 咽痛 |  |
| 嗅/味觉减退 |  |
| 腹泻 |  |
| 肌肉酸痛 |  |
| 嗜睡 |  |

- 相比上一次测试的时候，你感到自己现在的状态：

（ ）变得非常糟了

（ ）变糟了

（ ）有些变糟了

（ ）没变化

（ ）有些改善了

（ ）改善了

（ ）有了非常大的改善
