# Supplementary material for: Psychological changes in athletes infected with Omicron after return to training: fatigue, sleep, and mood
Source: PeerJ. 2023 Jun 15;11:e15580. doi: 10.7717/peerj.15580 (PMC10276985; doi:10.7717/peerj.15580)
Supplement: Supplemental Information 4 [file peerj-11-15580-s004.docx]

**COVID-19 Infection Symptoms Questionnaire（First Test）**

Name:_____ Team:_____

- Date of first negative results in nucleic acid/antigen test: __________
- Date of first negative results in nucleic acid/antigen test after infection: __________
- Date of return training: __________
- Did you have fever during the infection? Yes No
- If you chose "Yes" in the last question, please fill in the maximum temperature of fever: __________
- Did you have any of the following symptoms during the infection? (√)

| Dry cough |  |
| --- | --- |
| Fatigue |  |
| Sore throat |  |
| Decreased smell/taste |  |
| Diarrhea |  |
| Muscle aches |  |
| Sleepiness |  |

- Do you still have any of the following symptoms as of now? (√)

| Dry cough |  |
| --- | --- |
| Fatigue |  |
| Sore throat |  |
| Decreased smell/taste |  |
| Diarrhea |  |
| Muscle aches |  |
| Sleepiness |  |

- Compared to when you get your first negative result after infection, you feel that you are now:

( ) very much worse

( ) worse

( ) a little worse

( ) no change

( ) a little improved

( ) improved

( ) very much improved

**COVID-19 Infection Symptoms Questionnaire（Second/Third Test）**

Name:_____ Team:_____

- Do you still have any of the following symptoms as of now? (√)

| Dry cough |  |
| --- | --- |
| Fatigue |  |
| Sore throat |  |
| Decreased smell/taste |  |
| Diarrhea |  |
| Muscle aches |  |
| Sleepiness |  |

- Compared to the previous test, you feel that you are now:

( ) very much worse

( ) worse

( ) a little worse

( ) no change

( ) a little improved

( ) improved

( ) very much improved
